# Supplementary material for: Resilience among Turkish adolescents: A multi-level approach
Source: PLoS One. 2024 Jul 1;19(7):e0300165. doi: 10.1371/journal.pone.0300165 (PMC11216579; doi:10.1371/journal.pone.0300165)
Supplement: Attachment — Submitted filename: Response to Reviewers.docx [file pone.0300165.s001.docx]

| EDITOR | RESPONSE |
| --- | --- |
| Please ensure that your manuscript meets PLOS ONE's style requirements, including those for file naming. The PLOS ONE style templates can be found at | Revised |
| In the Methods ethics statement, you specified that verbal consent was obtained. Please provide additional details regarding how this consent was documented and witnessed, and state whether the IRB approved this. | Revised and explained in the methods ethics statement between the lines 225-237. |
|  |  |
| Your ethics statement should only appear in the Methods section of your manuscript. If your ethics statement is written in any section besides the Methods, please delete it from any other section. | Revised |
| Please review your reference list to ensure that it is complete and correct. If you have cited papers that have been retracted, please include the rationale for doing so in the manuscript text, or remove these references and replace them with relevant current references. Any changes to the reference list should be mentioned in the rebuttal letter that accompanies your revised manuscript. If you need to cite a retracted article, indicate the article’s retracted status in the References list and also include a citation and full reference for the retraction notice. | Revised. All the references have been double check. Besides newly edit references highlighted with yellow. |
| REVIEWER 1 | RESPONSE |
| Abstract  Provide more specific information about the findings. For example, instead of stating that physical exercise, emotion regulation, and self-efficacy are significant indicators of resilience, provide some numerical data or effect sizes to demonstrate the strength of these relationships. | Necessary explanations were made in the abstract between the line numbers 44-56 and highlighted in yellow. |
| Introduction  Some statements are quite broad and could benefit from more specific details or examples. For instance, when discussing the impact of emotional regulation on resilience, you mention "unreasonable and harmful behaviors." Providing specific examples or studies to illustrate this point could strengthen your argument. | Neccesary revisions made between the line numbers 90-92, 138-140, 157-163 with proper citations and and highlighted in yellow. |
| Hypotheses  The hypotheses formulated in the theoretical background section are relevant and logically derived from the literature. However, consider providing a brief justification or rationale for each hypothesis to clarify the expected relationships between variables. | The hypotheses have been revised to provide a clearer explanation of the relationships between concepts. |
| Introduction  Consider adding a brief concluding paragraph to summarize the main points discussed in the introduction and reiterate the significance of the study. This will provide closure to the section and reinforce the relevance of the research objectives. | Neccesary revisions made between the line numbers 176-181 and highlighted in yellow. |
| Method 1. Clarity and Organization: The section is well-organized and provides detailed information about each aspect of the study. However, consider breaking down lengthy paragraphs into smaller, more digestible chunks to improve readability. Bullet points or subheadings can be used to delineate different aspects of the methodology, making it easier for readers to follow the process. | The lengt of the paragraphs decreased and sucheadings were used. All the methods section revised according to the comments. |
| 2. The use of G*Power to determine the required sample size is appropriate and adds to the rigor of the study. However, it would be beneficial to provide a brief rationale for why a sample size of 439 participants was deemed sufficient for the study. Mentioning the expected effect size, significance level, and statistical power can help justify the chosen sample size. | Neccesary revisions made between the line numbers 295-301 and highlighted in yellow. |
| 3. The section on data collection procedures is clear and provides sufficient detail about the sampling method and ethical considerations. Consider briefly discussing any measures taken to ensure data quality and participant confidentiality, as these aspects are crucial in maintaining the integrity of the study. | A brief information given between the lines 225-237.. |
| 4. The description of each data collection tool is thorough and includes information about their development and validation. However, consider providing a brief rationale for why these specific scales were chosen for the study. Discussing how each tool aligns with the research objectives and measures the constructs of interest can enhance the justification for their selection. | Necessary information given between the lines 250-262, 266-272, 275-282 and 293-294. |
| 5. The description of data analysis procedures is detailed and includes information about the statistical tests used to analyze the data. Consider providing a rationale for why hierarchical regression analysis was chosen as the primary method for examining the relationship between independent and dependent variables. Additionally, mention any steps taken to address potential biases or confounding variables during data analysis. | Neccesary revisions made between the line numbers 308-310 and highlighted in yellow. |
| 6. It's commendable that assumptions for regression analysis were checked and met. However, briefly mentioning the steps taken to ensure the validity of the assumptions, such as normality, linearity, and multicollinearity, would enhance transparency and credibility. | Neccesary revisions made between the line numbers 314-318 and highlighted in yellow. |
| results  • Ensure precision in reporting statistical results, including regression coefficients, significance levels, and effect sizes. Clearly state the statistical tests used and provide appropriate degrees of freedom and p-values to facilitate accurate interpretation of the findings | Neccesary revisions made between the line numbers 346, 352, 354 and highlighted in yellow. |
| • Ensure consistency in the presentation of results across tables and graphs. For instance, use consistent labeling and formatting conventions to facilitate understanding and comparison of findings. Additionally, consider providing titles and legends for the graphs to clarify the variables being depicted. | Titles were given to the tables and graphs so that the findings could be clearly understood. |
| • The use of Jeremy Dawson's slopes to visualize the relationships between variables is informative. Consider providing a brief explanation of how the slopes were calculated and interpreted, particularly for readers who may not be familiar with this method. Additionally, discuss the implications of the observed slopes for understanding the relative influence of different variables on resilience | Neccesary revisions made between the line numbers 361-364, 380-381 and highlighted in yellow. |
| Discussion  1. Consider providing more context for the findings by discussing them in relation to the broader literature on adolescent resilience, physical activity, emotion regulation, and self-efficacy. Highlight how the current study contributes to existing knowledge and what new insights it offers to the field. | Suggested changes have been added with as per citations between the lines 394-396, 397-405, 410-421. |
| 2. Provide deeper interpretation of the results by discussing the underlying mechanisms that may explain the observed relationships between variables. For example, discuss why physical activity, emotion regulation, and self-efficacy are important predictors of resilience during adolescence, drawing on theoretical frameworks and empirical evidence. | Suggested changes have been added with the linse between 456-465. |
| 3. Discuss the practical implications of the findings for interventions aimed at promoting resilience among adolescents. Consider how the insights gained from the study can inform the design and implementation of programs and policies targeting physical activity promotion, emotion regulation skills training, and self-efficacy enhancement in adolescent populations. | practical implications section and new references have been added. 467-479, 613-619 |
| 4. Provide a thorough discussion of the study limitations and suggest directions for future research to address these limitations. Consider discussing potential methodological improvements, such as using longitudinal designs, implementing more rigorous sampling methods, or employing objective measures of physical activity. | Suggestions were made for further research 466-479. |
| 5. Acknowledge the limitations of generalizability inherent in convenience sampling and discuss how these limitations may impact the interpretation and application of the study findings. Consider discussing the potential differences between the study sample and the broader adolescent population and how these differences may affect the generalizability of the results. | “Limitations related to the generalisability of the data can be reduced by using probability sampling methods.” Added. 470-471. |
| 6. Conclude the discussion by summarizing the key findings, reiterating their significance, and emphasizing the implications for theory, practice, and future research. Provide a clear and concise summary of the main takeaways from the study and how they contribute to advancing knowledge in the field of adolescent resilience. | In paragraph 2 of the discussion section, general conclusions are given and an addition is made. |
| REVIEWER 2 | RESPONSE |
| The authors point out the subjective nature of the physical activity questions. I have no suggestions for change, but the percentage of the sample involved in physical activities was not high. In future research it might be valuable to attempt to recruit more, and in the analysis separate physical activities into team sport activities versus individual activities because of the different social contexts. A possible question for the future: ‘Would those involved in team sports be more likely to show resilience because they are working together as a team?’ | Thank you for this very valuable comment. We look forward to examining the answers to this question in terms of multiple variables in a similar study in the future. |
