# Supplementary material for: Resilience among Turkish adolescents: A multi-level approach
Source: PLoS One. 2024 Jul 1;19(7):e0300165. doi: 10.1371/journal.pone.0300165 (PMC11216579; doi:10.1371/journal.pone.0300165)
Supplement: Attachment — Submitted filename: Response to Reviewers.docx [file pone.0300165.s002.docx]

| EDITOR | RESPONSE |
| --- | --- |
| Please review your reference list to ensure that it is complete and correct. If you have cited papers that have been retracted, please include the rationale for doing so in the manuscript text, or remove these references and replace them with relevant current references. Any changes to the reference list should be mentioned in the rebuttal letter that accompanies your revised manuscript. If you need to cite a retracted article, indicate the article’s retracted status in the References list and also include a citation and full reference for the retraction notice. | Revised |
| REVIEWER 3 | RESPONSE |
| Statistical values (numbers) should be removed from the abstract | Revised |
| The theoretical structure of resilience is clearly emphasized in the introduction. | Neccesary revisions made between the line numbers 84-88 and highlighted in yellow. |
| How was sampling decided in the method? Must be explained, power analysis can be done | Sampling was decided by cross-sectional-convenience sampling method. It is highlighted in yellow on line 214. Additionally, G*Power analysis was performed. Highlighted in yellow between lines 312-315. |
| The inclusion and exclusion criteria for the study should be clarified | Neccesary revisions made between the line numbers 242-244 and highlighted in yellow. |
| The name of the institution should be blinded in the ethics section | Neccesary revisions made between the line numbers 240-241 and highlighted in yellow. |
| Articles and tables should generally be reviewed according to the journal writing rules. | Revised |
| The presentation of Table 2 is not appropriate, means and SDs should be shown in different places in Table 2. | Neccesary revisions made between the line numbers 349-350 and highlighted in yellow. |
| Confidence interval values should be added to Table 3, Durbin Watson values of the models should be presented. | Neccesary revisions made between the line numbers 356-357 and highlighted in yellow. |
|  |  |
